# Structuring telemonitoring in heart failure care in The Netherlands: design and operational protocol of a nationwide initiative

**DOI:** 10.1093/ehjdh/ztaf130

**Published:** 2025-11-05

**Authors:** Charell Jansen, Luuk C Otterspoor, Petra E J van Pol, Georges C IJff, Steef J Sinkeler, Mieke van den Heuvel, Martijn E P Jansen, Merel Scholten, Kim van Zutphen, Cindy C A G Verstappen, Nienke Hermanides, Ivo A Joosen, Stefan Heinen, Jeroen G Valk, Miriam M R Vollenbroek-Hutten, Mark J Schuuring, Gerardus P J van Hout

**Affiliations:** Department of Biomedical Signals and Systems, University of Twente, Drienerlolaan 5, Enschede 7522 NB, the Netherlands; Department of Cardiology, Medisch Spectrum Twente, Enschede, the Netherlands; Department of Cardiology, Catharina Hospital, Eindhoven, the Netherlands; Department of Cardiology, OLVG, Amsterdam, the Netherlands; Department of Cardiology, Maasstad Hospital, Rotterdam, the Netherlands; Department of Cardiology, Martini Hospital, Groningen, the Netherlands; Department of Cardiology, Medisch Spectrum Twente, Enschede, the Netherlands; Department of Cardiology, Catharina Hospital, Eindhoven, the Netherlands; Department of Cardiology, OLVG, Amsterdam, the Netherlands; Department of Cardiology, Canisius—Wilhelmina Hospital, Nijmegen, the Netherlands; Department of Cardiology, Catharina Hospital, Eindhoven, the Netherlands; Department of Cardiology, St. Antonius Hospital, Nieuwegein, the Netherlands; Department of Cardiology, Canisius—Wilhelmina Hospital, Nijmegen, the Netherlands; Department Better Together, Santeon, Utrecht, the Netherlands; Department of Biomedical Signals and Systems, University of Twente, Drienerlolaan 5, Enschede 7522 NB, the Netherlands; Department of Cardiology, Medisch Spectrum Twente, Enschede, the Netherlands; Department of Biomedical Signals and Systems, University of Twente, Drienerlolaan 5, Enschede 7522 NB, the Netherlands; Department of Cardiology, Medisch Spectrum Twente, Enschede, the Netherlands; Department of Biomedical Signals and Systems, University of Twente, Drienerlolaan 5, Enschede 7522 NB, the Netherlands; Department of Cardiology, Medisch Spectrum Twente, Enschede, the Netherlands; Department of Cardiology, St. Antonius Hospital, Nieuwegein, the Netherlands

**Keywords:** Heart failure (HF), Home telemonitoring system (hTMS), Remote monitoring, Hybrid care

## Abstract

**Aims:**

Home telemonitoring systems (hTMS) have demonstrated positive effects on hospitalizations and mortality for patients with heart failure (HF). However, there is a high degree of heterogeneity in the development and integration of hTMS. This limits the potential for large-scale impact and equitable access to high-quality remote care. Therefore, this manuscript describes a detailed protocol of a scalable high-volume hTMS system which has been standardized and implemented across seven large teaching hospitals in The Netherlands (*Graphical Abstract*).

**Methods:**

A multicentre, qualitative, stakeholder-inclusive approach was applied from the early stages of the hTMS protocol development. In January 2023, a HF-core team was established, comprising cardiologists, HF nurse specialists, and experts in implementation and process optimization, from all hospitals. The team developed unified protocols for remote monitoring of vital signs (blood pressure, heart rate, weight), including standardized cut-off values to generate automated alerts. In addition, protocols were established on how to rapidly titrate HF medication with the use of hTMS. A new department, the Medical Service Centre was established in each site to monitor these alerts and contact patients.

**Conclusion:**

This manuscript describes the development of the hybrid care pathway and the operational protocol of the hTMS. Future publications will elaborate on outcomes related to implementation success, clinical endpoints, and cost-effectiveness, which are currently being collected and evaluated as part of the programmes prospective design.

## Graphical Abstract

**Graphical Abstract:**
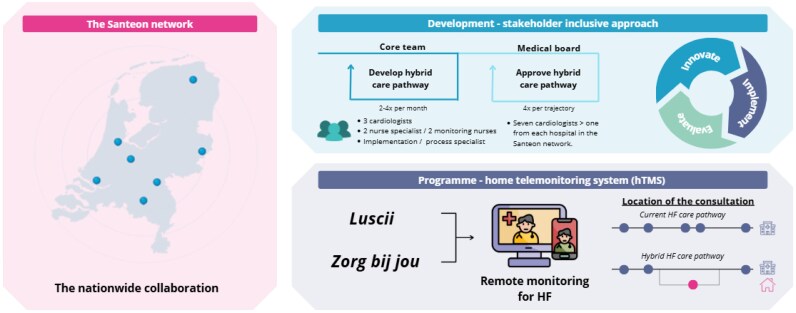
The Nationwide Network Participating in the Standardised hybrid care programme for Heart Failure

Key learning points

1. **What is already known**
  - hTMS could potentially improve clinical outcomes in HF, including reductions in hospitalizations and mortality in research settings.
  - Development and integration of hTMS into standard care is currently highly variable and often lacks uniformity. Real-life registries and data from clinical care systems is lacking.
2. **What this study adds**
  - This nationwide, multicentre programme demonstrates feasibility of standardizing hTMS across seven hospitals in a wide geographic area in the Netherlands, using stakeholder-driven development process.
  - Shows the scalability of a national, uniform hybrid care programme.
  - Offers a blueprint for broader implementation of hTMS, both for HF and other (chronic) diseases.

## Introduction

Heart failure (HF) is a progressive, chronic condition associated with high morbidity and mortality.^1,2^ The global prevalence of HF is estimated to be 64 million, including approximately 250.000 patients in the Netherlands. With HF-related annual admission rates of 34.000 and an average length of stay of seven days, HF places a considerable burden on the Dutch healthcare system.^3^

The growing prevalence of HF, combined with labour intensive treatment options, imposes a substantial demand on healthcare resources.^4^ This directly influences patient Quality of Life, risk of (re-)admission and prognosis.^1,5^ To ensure sustainable and effective management, innovative solutions are required.^4^ Remote patient management, including home telemonitoring systems (hTMS), are increasingly being used to address these challenges. The patient is given control over their illness: independently, if possible digitally at home and only in the hospital if necessary. With the shift from hospital based to home-based care with the use of hTMS, a hybrid care model has been developed. In the Dutch healthcare system, telemonitoring is reimbursed by health insurance, enabling hospitals to integrate telemonitoring as part of routine care. Embedding hTMS within the existing standard of care is critical to ensure the programme functions as a supportive tool rather than an additional burden.^6,7^

Despite the growing adoption of hTMS, implementation is often fragmented, lacking uniform standards or structured integration into existing care pathways.^8^ This lack of standardization presents a critical gap: without a unified approach, the quality and accessibility of remote HF care may vary significantly, thereby limiting its potential and raising concerns about equitable care delivery.^4^ The starting point is not technology, but the redesign of care processes, including an explicit role of the hTMS. Therefore, a group of stakeholders developed a programme to standardize telemonitoring for patients with HF on a national level while remaining compatible with existing care structures.

This paper describes this process, carried out across seven large Dutch hospitals, detailing the collaborative design process, the agreed-upon workflows, and medical protocols for the integration of hTMS into conventional healthcare (*Graphical Abstract*). The primary aim of this programme is improving patient self-management, increasing care efficiency in the context of growing HF prevalence and staff shortages, and exploring the feasibility of integrating telemonitoring as part of standard chronic HF care.^9^ While future work will focus on the implementation process, evaluation of clinical outcomes, cost-effectiveness, patient-reported outcomes and implementation facilitators and barriers, the present manuscript focuses on the rationale, development, and practical integration of the programme into routine care.

## Methods

### Design of the programme

#### Participating hospitals

In the Netherlands, seven teaching hospitals form a consortium since 2007, the Santeon network (*Graphical Abstract*). The aim of the consortium is to continuously improve the quality of medical care through open collaboration and innovation. The affiliated hospitals have a key role in HF care across a wide geographic area, collectively serving over one in ten patients nationwide and representing approximately 12% of the total hospital care in the Netherlands.^10^ This network comprises Canisius hospital (Nijmegen), Catharina hospital (Eindhoven), Maasstad hospital (Rotterdam), Martini hospital (Groningen), Medical Spectrum Twente (Enschede), OLVG (Onze Lieve Vrouwe Gasthuis) hospital (Amsterdam) and St. Antonius hospital (Nieuwegein and Utrecht).^11^

#### Stakeholder-inclusive approach

To ensure standardization and uniformity, a multicentre, qualitative, stakeholder-inclusive approach was applied from the early stages of programme development (*Table 1*). A multidisciplinary HF-core team consisting of HF cardiologists, HF nurse specialists, and experts in process optimization and implementation was established at the start of the development process (*Graphical Abstract*). In addition, a medical board was created, comprising one HF cardiologist and HF nurse specialist from each participating hospital, supported by a central programme coordinator. Multiple supporting workstreams were established to facilitate structured input across all domains of the programme, including dedicated teams for data infrastructure and evaluation, app development and technical support. The stakeholders were identified based on their (in)direct involvement in HF care or their practical and strategic roles in the implementation of the hTMS.^12,13^

**Table 1:** Involved stakeholders during development of the hTMS

	Stakeholder-inclusive approach	
	HF core team	Medical board
**Members**	Three cardiologists Three HF nurse specialist One monitoring nurse Process- and implementation specialist	Seven cardiologists (one from each Santeon site)
**Role**	Develop, evaluate and further develop a uniform, hybrid working method, in line with intended impact objectives.	Approval of uniform, hybrid working method as drawn up by the core team.
**Meeting frequency**	Two to four times per month	± once each three months.
**Involvement in the development process**	Entire process: care pathway development, implementation, evaluation and further development.	

Patients were not directly represented as a stakeholder during the earliest stages of development. However, their perspectives were indirectly included. First, the feedback given through the application was used iteratively to improve the in-app workflow and user experience. Second, the hTMS initiative is fuelled by a patient centred organization. Third, the protocol is under review by a patient federation. The structured integration of patient feedback has been a valuable contributor to the iterative optimization of the programme. Adjustments to protocols, workflows, and patient communication strategies were made based on real-world insights and user needs.

This stakeholder-inclusive design is based on a synthesis of scientific evidence, expert consensus (clinical knowledge), and emerging best practices across multiple centres to ensure a unified and evidence-based approach to care^12–15^

#### The development process

**Figure 1:**
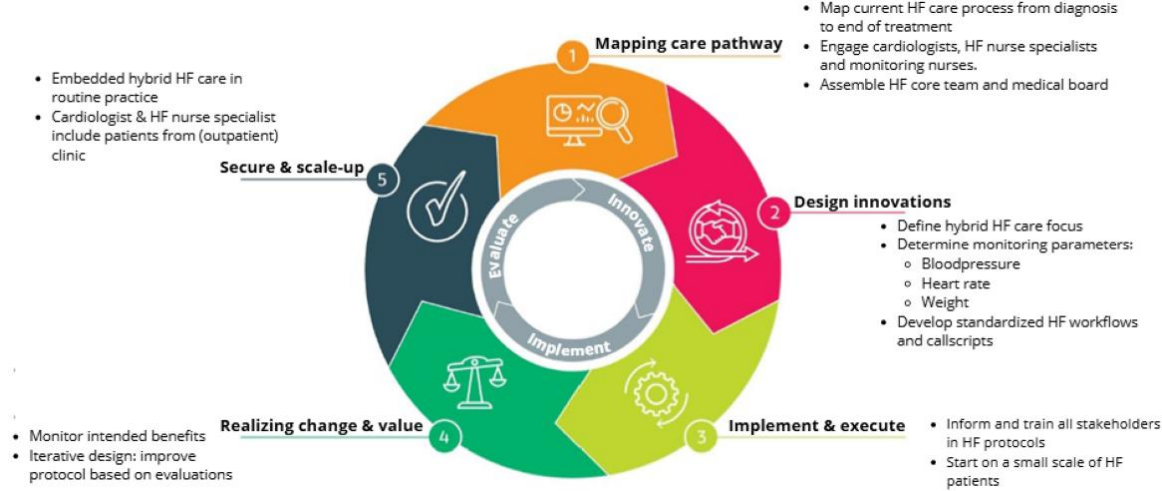
Stakeholder-inclusive development cycle. https://samendezorgvernieuwen.nl

The design of the care process was guided by an iterative development cycle, incorporating principles of all stakeholders (*Figure 1*). During the first year, weekly meetings were held with the HF-core team, where the process of development was discussed. From the second year onward, biweekly meetings with the medical board were held, to ensure applicability of the protocols in all sites. Each cycle included phases of co-design, prototyping, testing, risk assessment and evaluation, allowing for continuous refinement based on real-world feedback.

### The programme

**Figure 2:**
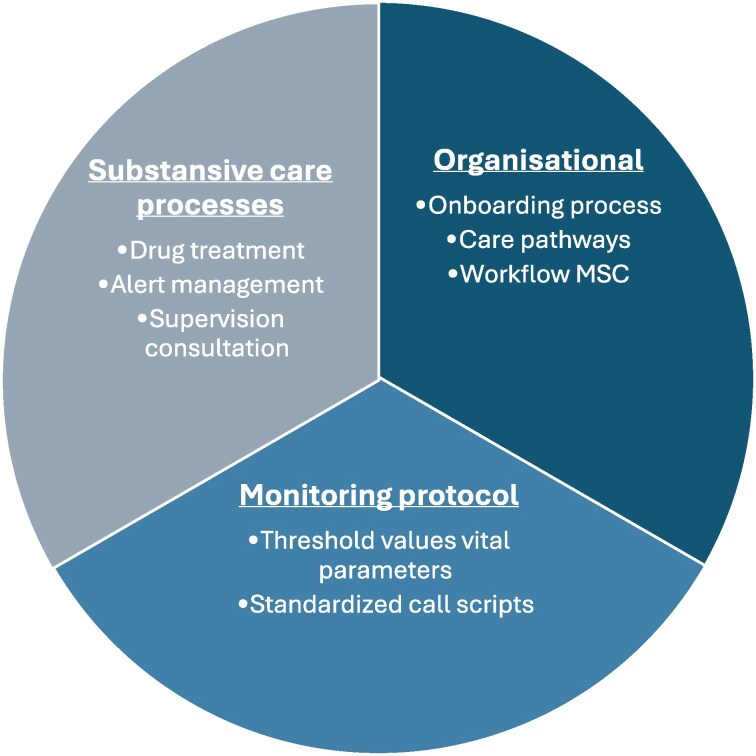
Components of the programme.

The seven Santeon hospitals created a collaborative, non-profit healthcare transformation programme and hTMS platform Zorg bij jou. The programme is a combination of an organizational structure, monitoring protocol and defined substantive care processes (*Figure 2*). A fundamental component of the programme is the digital platform Zorg bij jou, designed with the aim to realize appropriate care. Digitally and at home whenever possible, and in the hospital when necessary. With this development, Santeon is a leader in digital healthcare transformation in the Netherlands. The initial Santeon consortium itself, however, was not designed or limited to promoting hTMS in HF care. The transformation of HF care is part of an overall transformation in the Dutch healthcare. The principles for this programme are standardization, digitalization, scalability and broad applicability.^16^

As part of this digital platform, remote patient management with the use of hTMS application, asks patients to measure their heart rate (HR), blood pressure (BP) and weight at home. With the use of an application called Luscii (Luscii Healthtech B.V.), the measurements are sent to and monitored in the hospital. Within the application, the measurements are supplemented by structured questionnaires assessing HF-related symptoms. Additionally, animated short educational modules are available about HF pathophysiology, lifestyle modifications, and pharmacotherapy with the aim to improve the self-management of patients by increasing disease-specific knowledge.

#### Population

Participation in telemonitoring is offered to all HF patients who meet the inclusion criteria (*Table 2*), without being mandatory. The decision to start using hTMS is made using shared decision-making. Patients who do not want or are unable to participate continue to receive the current standard of care (in-person visits and/or telephone consultation) by HF nurse practitioners at the dedicated HF outpatient clinic. To enhance accessibility, the application was deliberately designed with a strong emphasis on ease of use, ensuring applicability even for individuals with limited digital literacy. It employs clear visualizations and an intuitive layout. Active strategies are used to support digital engagement among patients. These include guidance during onboarding for the patient in the hospital: support by installing the application and providing help with measurements by HF nurses or front-office staff from the MSC as well as providing the app in multiple languages and/or involving informal or family caregivers in the process.

**Table 2:** In- and exclusion criteria

Inclusion criteria
The patient: is (newly) diagnosed with HF; is >18 years of age; possesses a smartphone or tablet on which the Thuismeten app can be installed; (or a caregiver willing to assist) has sufficient digital literacy to operate the smartphone and app; (or a caregiver willing to assist) is proficient in the Dutch language, as long as the app is not yet available in other languages; has access to a weighing scale and BP monitor (or intends to acquire these).
**Exclusion criteria**
Patients who are not motivated or willing to participate in home monitoring. Patients with end-stage HF or marginal clinical condition.

#### Onboarding process

Patients eligible for the program can be onboarded in the clinic when discharge is approaching, at the outpatient clinic and at the first cardiac aid or emergency department. The hTMS operates within two structured protocols (stable/unstable), designed to accommodate patients based on their clinical stability. The criteria defining each protocol are outlined in *Table 3*. During the onboarding phase (see Supplementary materials  *S1*), the HF nurse practitioner or cardiologist provides the MSC nurse with essential patient-specific information, including the patients target weight (weight at euvolaemia) and which protocol the patient should be enrolled into.

**Table 3:** Clinical protocols for remote monitoring

Stable protocol	Unstable protocol
Patient is on OMT for at least 1 month. OMT is based on the most recent ESC guidelines for the diagnosis and treatment of acute and chronic HF.	Newly diagnosed with HF (HF *de novo*).
Patient is euvolaemic and therefore, in addition to OMT, well set on diuretics, without adjustments in dosage during the past month.	Patient had been (re)admitted for HF.
There is no change in complaints and symptoms during the past month.	Exacerbation of HF that requires changes in medication, including diuretics.
Patient has sufficient clinical knowledge of HF and the early detection of symptoms of worsening HF.	Patient is not yet on OMT.
Patient applies adequate self-management and seeks timely contact in case of complaints.	Patient is symptomatic, anxious and/or uncertain.
	Patient still needs help/guidance in the early detection of symptoms of worsening HF.

#### Unstable protocol

Patients are enrolled into the *unstable protocol* 1) prior to hospital discharge (due to congested HF), 2) during an initial visit at the HF outpatient clinic (rapid sequencing of medication) or 3) an ambulant exacerbation for which increasing oral diuretics are required (see Supplementary materials  *S2*).

The unstable protocol comprises an intensive monitoring phase in which patients track and fill in the vital parameters from Monday through Friday and complete the Recognizing complaints questionnaire once per week. The telemonitoring is limited to weekdays. Patients are clearly informed about the telemonitoring not being a 24/7 service and that clinical responsibility outside telemonitoring hours, including evenings, nights, and weekends, remains with the regular care system. This means, in case of clinical deterioration outside weekdays and office hours, patients need to contact the HF outpatient clinic, the emergency department, or emergency services (112) as appropriate. This protocol is aligned with standard care procedures for HF patients and ensures patient safety while maintaining a feasible workload for the monitoring team.

The goal is 1) Ensuring disease stability directly after hospital admission, 2) achieving personalized optimal medical therapy (OMT) based on the most recent European Society of Cardiology (ESC) guidelines as efficiently as possible or 3) decongest patients with congestion that do not require hospital admission. OMT is defined as the initiation of all pharmacological classes recommended by current HF guidelines (RAS-inhibitor, beta-blocker, MRA, and SGLT2 inhibitor, depending on type of HF based on left ventricular ejection fraction), with up-titration to the highest tolerated dose whenever possible. Therapy is considered optimal if all classes were at least attempted, unless contraindicated or not tolerated.^2^

#### Stable protocol

Patients who have achieved OMT and demonstrate clinical stability over a period of two to four weeks are eligible for enrolment in the *stable protocol*. Clinical stability is defined as the absence of worsening symptoms, no further need for medication adjustments, and confirmed euvolaemia, as evidenced by stable body weight and the absence of signs of congestion. Patients may enter the stable protocol either directly from the outpatient clinic or through transition from the unstable protocol, following a sustained period of clinical and haemodynamic stability.

The stable protocol enables long-term remote surveillance, reducing the need for frequent in-person visits while maintaining or even improving the high standards of patient safety and disease control. Patients enter vital signs and symptom assessments at least once per week. The primary goal is preventing HF deterioration through early detection and intervention.

#### Transition between stable and unstable protocol

**Figure 3:**
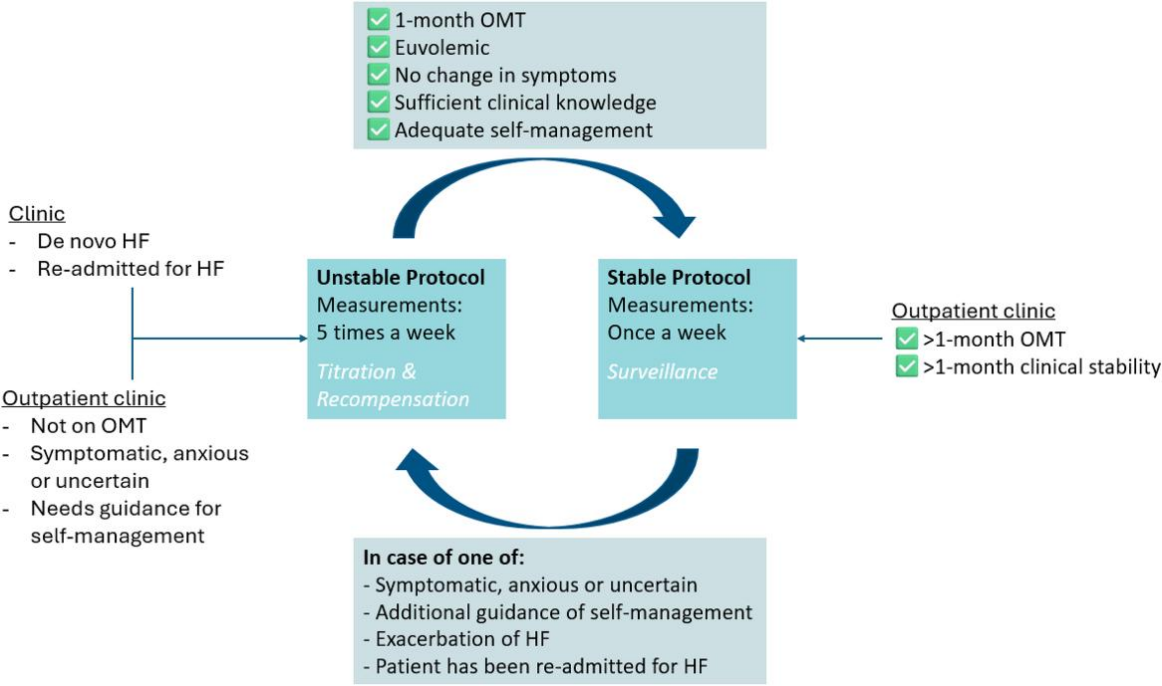
Flow between unstable and stable protocol.

Transition between these protocols is dynamic: patients in the stable protocol may be escalated to the unstable protocol if they experience symptom worsening, weight gain due to decompensation or have been (re)admitted to the hospital. Conversely, patients in the unstable protocol may transition to the stable protocol in case of stability, defined by reaching OMT, stable weight, and no need for diuretic adjustments. See *Figure 3* for a visualization.

An important indicator is the biomarker NT-proBNP since it significantly aids in assessing fluid status remotely.^17^ Patient-specific levels in time have been shown to strongly correlate with volume status.^18,19^ Detailed data on the moments of measurements and correlation with volume status are provided in Supplementary materials  *S3*.

#### Duration of telemonitoring—offboarding

Given the chronic nature of HF, patients are expected to remain in the programme as long as they stay engaged and derive clinical benefits as judged by the secondary care providers (cardiologists and HF nurses). Offboarding from the programme may be considered in cases of sustained patient inactivity, defined as failure to complete scheduled measurements and lack of response to outreach by monitoring nurses. If such inactivity persists for one month, a final telephone contact attempt is made. In the absence of any response or identifiable reason, the patient withdraws from the programme.

### Medical service centre

**Figure 4:**
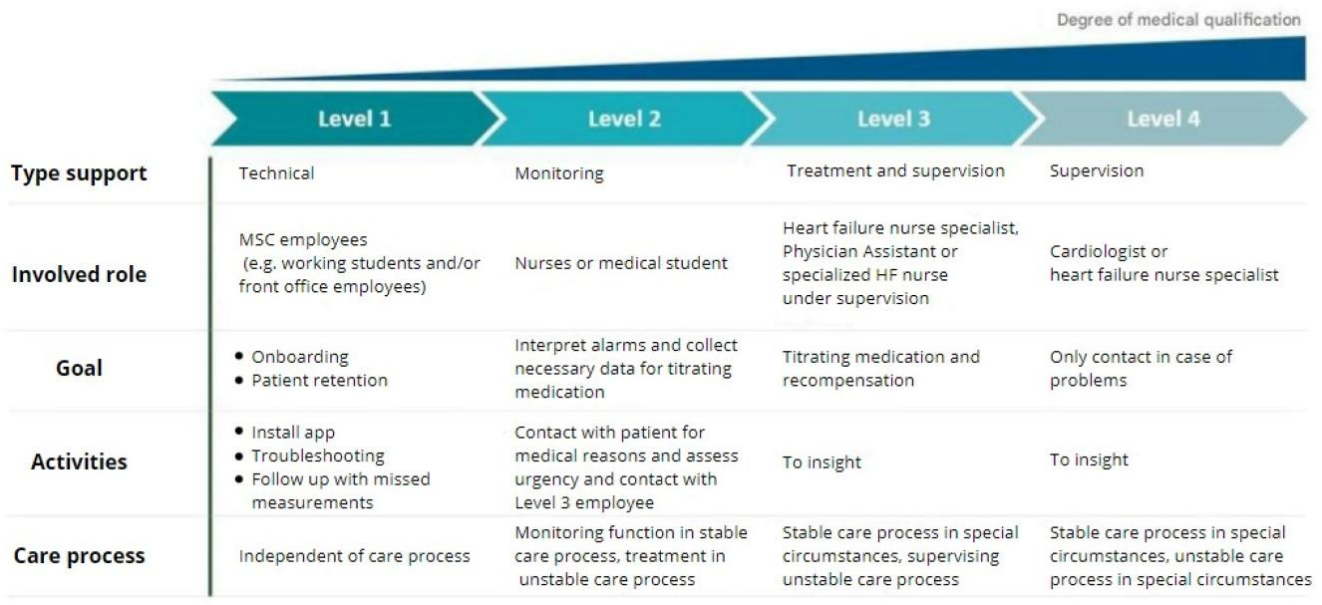
Support levels medical service centre.

In each of the seven participating hospitals, a new department has been set up: the Medical Service Centre (MSC). In this department, the remotely measured vital parameters are monitored. The monitoring process is a collaborative effort involving, in growing degree of medical qualification: technical support staff, monitoring nurses, HF nurse specialists, and cardiologists (*Figure 4*). The technical support staff (level 1), support in the onboarding of new patients and help patients with installation of the application. The role of the monitoring nurse (level 2) varies between the stable and unstable protocols. During the unstable phase, the monitoring nurse actively contacts patients to enable changes in treatment. During the stable phase, their role is limited to monitoring. The monitoring nurses are supervised by HF nurse specialists (level 3). They provide expert guidance, and titrate guideline-directed medical therapy as well as diuretics. They also decide on individualization of cut-off values of BP, weight and HR and protocol transition. To ensure structured oversight, a planned supervision meeting is held between level 2 and 3 staff at least once per week. The frequency of these meetings may vary depending on hospital-specific factors and the number of patients in telemonitoring. In centres with a small monitoring population, questions about individual patients can typically be managed with a non-planned phone call. However, as the number of patients increases, so does the volume of cases to be discussed. In those cases, regular, scheduled meetings are necessary to ensure structured oversight and be sure there is time to discuss all cases. Only in case of a complex situation requiring additional clinical expertise or when complications arise, the cardiologists (level 4) will be involved.

#### Alert management

The programme includes a monitoring protocol in which threshold values are established for the vital parameters (*Table 4*). These threshold values are agreements of the medical board in line with the HF treatment guidelines.^2^ In the case the entered value exceeds the limit, an alert will be sent to the MSC (see Supplementary materials  *S4*).

**Table 4:** Threshold values for alert management

Vital parameter	Status measurement	Threshold value
**Blood pressure (BP)**	Sudden increase Moderate *hypertension* Severe *hypertension* *Hypotension*	>30 mmHg 160–179/100–109 mmHg > 180/>110 mmHg < 90/60 mmHg
**Heart rate (HR)**	Frequency Frequency	≤50/min (rest) ≥ 100/min (rest)
**Weight**	Weight compared to last measurement Measurement below or above target weight	+ or—1.5 kg ≥ 2 kg

In the event of an alert, patient contact can be established through three modalities. First, the MSC nurse can send a text message via the application to guide the patient in responding to abnormal values. Second, the patient can proactively contact the MSC by phone. Third, the MSC nurse may initiate a phone call for a more in-depth assessment of the patients symptoms. To enhance standardization, detailed call scripts, developed by the HF-core team, have been integrated into the programme (see Supplementary materials  *S2*). In case of recurrent threshold deviation without significant symptoms or risk of worsening HF, cut-off values may be individualized according to advice of the level 4 staff.

### Substantive care processes

As previously described, the level 2 employees (MSC nurses), in addition to monitoring, also play a role in the treatment of patients in the unstable protocol. Regular medication adjustments in combination with telephone consultations with a level 2 employee (every 7–14 days) in order to achieve rapid and structured OMT in patients within the unstable protocol.^2^ For this titration standardized titration schedules have been developed (see Supplementary materials  *S5*).

As part of the standardized approach, diuretics are used to manage congestion and fluid overload, ensuring symptomatic relief. The dosage can be adjusted based on weight fluctuations, following a standardized diuretic escalation protocol designed to guide these adaptations (see Supplementary materials  *S2*).

### Inclusions

From September 2023, the implementation of the developed hTMS programme commenced across all participating hospitals. To ensure consistency and adherence to the standardized approach, implementation was guided and closely monitored by the HF-core team. As the programme is covered by basic health insurance and designed to become an integral part of standard care, patient enrolment remains ongoing.

The hTMS was further refined during the inclusion period based on stakeholder feedback, practical experiences, and iterative improvements. While this reflects real-world implementation, such changes may influence outcomes, particularly when assessing effectiveness or implementation success. To address this analytically, all major changes to the programme (e.g. protocol adjustments or workflow optimizations) are being systematically documented, including their timing. This will be integrated in the analysis by evaluating temporal effects in future analyses by incorporating time-dependent covariates or stratifying analyses by implementation phase.

### Planned evaluation and analysis

**Figure 5:**
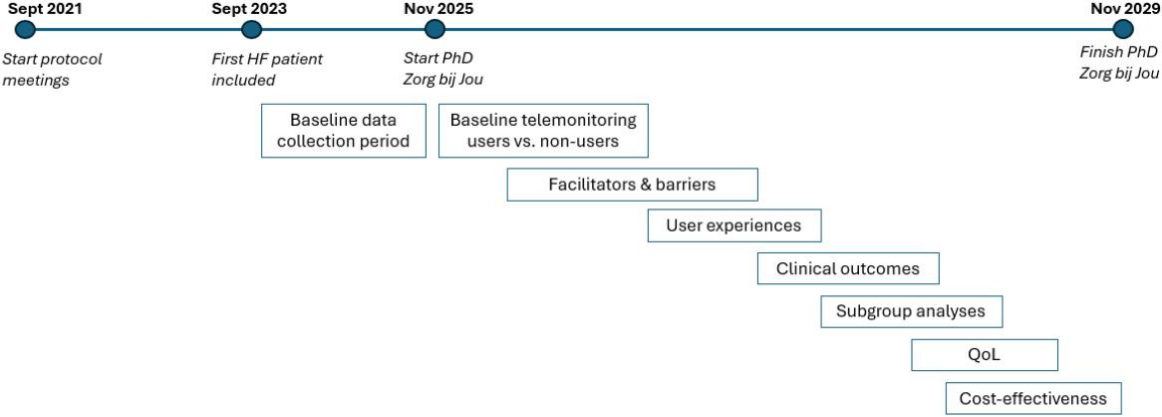
Schematic overview of the planned studies hTMS in HF care.

To evaluate the impact and feasibility of the hTMS, several analyses are currently being developed and conducted (see *Figure 5* for the timeline). First, baseline characteristics of patients enrolled in the hTMS will be described and compared to those not (yet) in telemonitoring. Particular attention will be given to medication optimization, as time-to-initiation and achievement of guideline-directed medical therapy. Subsequently, the effect on clinical outcomes, including hospitalizations and mortality will be analysed. Subgroup analyses will explore differential effects across patient populations, and a cost-effectiveness analysis will assess the economic implications of the programme. In addition, studies focusing on the implementation processes will investigate facilitators and barriers encountered during the roll-out phase. User experience will be evaluated from both the patient and healthcare provider perspective, and finally, analyses will be conducted to understand participation dynamics, including reasons for drop-out and non-enrolment, linked to patient characteristics and motivations.

Together, these studies aim to provide a comprehensive evaluation of the clinical effectiveness, cost-efficiency, and implementation feasibility of the hTMS in routine clinical practice.

## Discussion

This manuscript outlines the protocol for the first nationwide, multicentre, standardized integration of hTMS into HF care in the Netherlands. The initiative represents a significant advancement in the delivery of chronic HF care, aiming to transit from a hospital-based model to a hybrid model of care. Embedding telemonitoring within the conventional structure of HF management is a crucial step in aligning practice with contemporary standards and harnessing digital tools to improve outcomes.

The programme, developed as an open, non-profit initiative, was intentionally designed to facilitate broader implementation beyond the Santeon network. By providing a structured, scalable and consistent framework across institutions, which is compliant with the latest ESC guidelines, this programme offers an operational model for broad implementation. The planned expansion of the hTMS system to two hospitals outside the consortium by the end of 2025 reflects this commitment to nationwide scalability and standardization of hTMS in HF care. Importantly, the open-source design allows for replication and adaptation in other healthcare systems, potentially serving as a blueprint for future integration of digital health tools into routine care elsewhere. The deliberate inclusion of a large, non-preselected patient cohort enhances generalizability and strengthens the foundation for widespread adaptation of hybrid HF care.

A significant strength of this programme lies in the cooperation of a major Dutch hospital consortium, the Santeon network. This alignment and standardization across multiple centres is key and can reduce variability in methods, a barrier to scalable telemonitoring internationally^7,20,21^ Although the efficacy of hTMS has been demonstrated, substantial heterogeneity in methods remains. Various studies showed, that while the efficacy of hTMS was demonstrated, the diversity in methods is considerable and several authors suggested that successful clinical implementation requires future standardization.^7,20^

Within the Dutch healthcare system, Santeon hospitals have a leading role in implementing value-based healthcare strategies. Nevertheless, a collaboration on this level is not without limitations. Achieving consensus among stakeholders from seven hospitals, each with distinct clinical cultures and digital infrastructures, proved time-consuming. While this collaborative structure may not be present in all hospital systems, the underlying principles, such as stakeholder involvement, iterative protocol design, and outcome-focused monitoring can be adapted to other settings or countries. Moreover, the challenges addressed (e.g. staff shortages, increasing HF prevalence, need for better GDMT titration) are widely shared, supporting the international relevance of our approach. In addition, similar hTMS have been introduced in other countries, often on a smaller scale or within research contexts, demonstrating international interest and growing momentum for broader integration of remote care models^7,22–24^

In the Netherlands, the healthcare providers, including cardiologists and HF nurses, took the lead in shaping the system around existing clinical workflows. In contrast to other European countries, where programmes are generally designed using a top-down structure, this bottom-up design promoted feasibility and clinical ownership. While long-term success will still depend on top-down support through sustainable funding and organization. Though not directly transferable to other health care system, the Dutch model illustrates broadly relevant principles: embedding innovation in existing care structures and placing health care professionals at the centre of design increases the likelihood of effective and sustainable implementation.

The potential benefits of the hTMS are multifaced. For patients, hTMS can provide reassurance, improve symptom monitoring, and support adherence to GDMT. From a healthcare system perspective, early identification of clinical deterioration can lead to timely interventions, reduce hospital admissions, and promote more efficient use of resources. In addition, the programme allows for more flexible resource allocation by integrating telemonitoring into structured workflows, which can help mitigate the growing pressure on specialized HF care. Future research should aim at providing answers if these proposed effects are seen in this real-life.

Although the added value of telemonitoring on preventing hospital admittance is currently only proven for NYHA class II–III patients post-hospitalization,^2^ the programme was designed to include all symptomatic patients with HF, except patients in the palliative phase. This pragmatic approach reflects real-world HF care and helps assess feasibility and scalability beyond the populations studied in prior RCTs. The goal of the system is not merely to provide care of superior quality but also from a societal perspective to be more cost-effective in the treatment and follow-up of HF patients. In addition, this allcomers approach also enables future subgroup analyses to evaluate which patient groups derive the greatest benefit from telemonitoring and if cost-effectivity truly increases thereby hopefully complementing existing RCT data.

A key limitation is the absence of clinical outcome data, economic analyses and patient-reported measures of the system in a RCT prior to real world use. However, as described in the methods, the programme will be evaluated with several qualitative and quantitative analyses. Several meta-analyses and randomized controlled trials have been done which support the clinical benefits of telemonitoring in HF care. The TIM-HF2 trial (Koehler *et al*., 2018)^9^ showed that remote patient management reduced all-cause mortality and cardiovascular hospitalizations. Scholte *et al*. (2023), conducted a comprehensive meta-analysis comprising 92 studies with over 36 000 HF patients, demonstrating that hTMS was associated with significant reductions in all-cause mortality (16%), first HF hospitalization (19%), and total HF hospitalizations (15%) compared to standard care.^7^ However, heterogeneity in study design and implementation limited the generalizability of these findings. Similarly, in a recent editorial, Koehler *et al*. highlight the ongoing challenges in standardizing hTMS and integrating them into routine clinical practice.^25^ BEAT-HF (Ong *et al*., 2016)^26^ and Tele-HF (Chaudhry *et al*., 2010)^27,28^ have yielded mixed results, largely due to differences in adherence, patient selection, and integration with existing care processes. Despite growing evidence from clinical trials, large-scale, real-world data on the long-term implementation and outcomes of telemonitoring in HF remain scarce. These findings underscore the need for harmonized, well-structured implementations to translate clinical efficacy into real-world effectiveness.

In addition to clinical benefits, telemonitoring is also expected to have an economic impact by reducing healthcare costs related to HF. Although several cost-effectiveness analyses of telemonitoring interventions in cardiovascular disease (under which HF) have been performed, these studies were typically conducted in research settings or on relatively small scales.^29,30^ In most cases, telemonitoring was implemented as a stand-alone intervention rather than integrated into routine care. Nevertheless, the results of these studies have shown promising outcomes. This programme aims to build on this evidence by analysing this integrated telemonitoring in a real-world HF care. This approach allows for a more accurate assessment of the feasibility, scalability, and economic impact of telemonitoring when adopted in another country with a possible different healthcare system.

This manuscript primarily aims to describe the development and implementation of a standardized hTMS protocol. Achieving consensus for a standardized integration of hTMS into routine care was considered a crucial first step to overcome variability in methods. Uniform methods provide the basis for uniform data collection and analysis. Although RCTs remain the gold standard for evaluating efficacy, the standardization of this programme was a crucial first step.^7,20^ Future research will elaborate on outcomes related to implementation, clinical endpoints, and cost-effectiveness, which are currently being collected and evaluated as part of the programmes prospective design.

## Conclusion

This manuscript describes a protocol of a nationwide, standardized programme to integrate hTMS into HF care. This initiative illustrates a structured and collaborative approach to implementing hTMS in HF care at a national level, with promising feasibility and scalability. The successful alignment across seven Santeon hospitals, covering a broad geographical area within the Netherlands, provides an important indication that this approach may be generalizable beyond a single hospital or region. Evaluation is ongoing to assess outcomes related to implementation success, clinical impact, and cost-effectiveness.

## Supplementary Material

ztaf130_Supplementary_Data

## Data Availability

Data will be available upon reasonable request to the Executive Committee Chair, with the approval of the oversight committee.

## Lead author biography

Charell Jansen, MSc, has a background in Health Sciences and is currently a PhD candidate at the cardiology department of Medisch Spectrum Twente and the University of Twente. Her research focuses on the use of digital health interventions to optimize guideline-directed medical therapy in patients with heart failure. She is particularly interested in how digital clinical decision support can be used to improve medication prescription and clinical outcomes.

Her academic interests include eHealth, quantitative clinical research and cost-effectiveness.

## References

[1] Savarese G, Becher PM, Lund LH, Seferovic P, Rosano GMC, Coats AJS. Global burden of heart failure: a comprehensive and updated review of epidemiology. Cardiovasc Res 2023;118:3272–3287. 35150240 10.1093/cvr/cvac013

[2] McDonagh TA, Metra M, Adamo M, Gardner RS, Baumbach A, Böhm M, et al 2023 focused update of the 2021 ESC guidelines for the diagnosis and treatment of acute and chronic heart failure: developed by the task force for the diagnosis and treatment of acute and chronic heart failure of the European Society of Cardiology (ESC) with the special contribution of the Heart Failure Association (HFA) of the ESC. Eur Heart J 2023;44:3627–3639.37622666

[3] Actuele cijfers hart- en vaatziekten . *Hartstichting*. https://www.hartstichting.nl/hart-en-vaatziekten/cijfers-hart-en-vaatziekten (17 March 2025)

[4] Steiner B, Neumann A, Pelz Y, Ski CF, Hill L, Thompson DR, et al Challenges in heart failure care in four European countries: a comparative study. Eur J Public Health 2023;33:448–454. 37164632 10.1093/eurpub/ckad059 PMC10234648

[5] Rao SK, Kimball AB, Lehrhoff SR, Hidrue MK, Colton DG, Ferris TG, et al The impact of administrative burden on academic physicians: results of a hospital-wide physician survey. Acad Med 2017;92:237–243. 28121687 10.1097/ACM.0000000000001461

[6] Schuuring MJ, Treskes RW, Castiello T, Jensen MT, Casado-Arroyo R, Neubeck L, et al Digital solutions to optimize guideline-directed medical therapy prescription rates in patients with heart failure: a clinical consensus statement from the ESC Working Group on e-Cardiology, the Heart Failure Association of the European Society of Cardiology, the Association of Cardiovascular Nursing & Allied Professions of the European Society of Cardiology, the ESC Digital Health Committee, the ESC Council of Cardio-Oncology, and the ESC Patient Forum. Eur Heart J Digit Health 2024;5:670–682. 39563907 10.1093/ehjdh/ztae064 PMC11570396

[7] Scholte NTB, Gürgöze MT, Aydin D, Theuns DAMJ, Manintveld OC, Ronner E, et al Telemonitoring for heart failure: a meta-analysis. Eur Heart J 2023;44:2911–2926. 37216272 10.1093/eurheartj/ehad280 PMC10424885

[8] Talal AH, Dharia A, Markatou M, Brown LS Jr, Bossert KE, Grubbs Z, et al Facilitated telemedicine as a patient-centered, sociotechnical intervention to integrate hepatitis C treatment into opioid treatment programs and overcome the digital divide among underserved populations: qualitative study. JMIR Public Health Surveill 2025;11:e68854. 40669057 10.2196/68854 PMC12286564

[9] Koehler F, Koehler K, Deckwart O, Prescher S, Wegscheider K, Kirwan B-A, et al Efficacy of telemedical interventional management in patients with heart failure (TIM-HF2): a randomised, controlled, parallel-group, unmasked trial. Lancet 2018;392:1047–1057. 30153985 10.1016/S0140-6736(18)31880-4

[10] Feiten en cijfers . *Santeon*. https://santeon.nl/over-santeon/feiten-en-cijfers/. (15 February 2025).

[11] Homepage . *Santeon*. https://santeon.nl/ (14 February 2025).

[12] Recabarren Silva J, Wu R, Scholes-Robertson N, Hughes A, van Zwieten A, Wong G, et al Reporting the involvement of patients and caregivers in identifying and designing healthcare interventions: the IDEAS framework. J Clin Epidemiol 2025;183:111784. 40216341 10.1016/j.jclinepi.2025.111784

[13] Fischer M, Safaeinili N, Haverfield MC, Brown-Johnson CG, Zionts D, Zulman DM. Approach to human-centered, evidence-driven adaptive design (AHEAD) for health care interventions: a proposed framework. J Gen Intern Med 2021;36:1041–1048. 33537952 10.1007/s11606-020-06451-4 PMC8042058

[14] Koole MAC, Kauw D, Winter MM, Dohmen DAJ, Tulevski II, de Haan R, et al First real-world experience with mobile health telemonitoring in adult patients with congenital heart disease. Neth Heart J 2019;27:30–37. 30488380 10.1007/s12471-018-1201-6 PMC6311159

[15] Bleijenberg N, de Man-van Ginkel JM, Trappenburg JCA, Ettema RGA, Sino CG, Heim N, et al Increasing value and reducing waste by optimizing the development of complex interventions: enriching the development phase of the Medical Research Council (MRC) framework. Int J Nurs Stud 2018;79:86–93. 29220738 10.1016/j.ijnurstu.2017.12.001

[16] Zorg bij jou . *Santeon.* available from https://santeon.nl/project/zorg-bij-jou/ (14 February 2025.

[17] Adamo M, Pagnesi M, Mebazaa A, Davison B, Edwards C, Tomasoni D, et al NT-proBNP and high intensity care for acute heart failure: the STRONG-HF trial. Eur Heart J 2023;44:2947–2962. 37217188 10.1093/eurheartj/ehad335

[18] Bayes-Genis A, Docherty KF, Petrie MC, Januzzi JL, Mueller C, Anderson L, et al Practical algorithms for early diagnosis of heart failure and heart stress using NT-proBNP: a clinical consensus statement from the Heart Failure Association of the ESC. Eur J Heart Fail 2023;25:1891–1898. 37712339 10.1002/ejhf.3036

[19] Brunner-La Rocca H-P, Sanders-van Wijk S. Natriuretic peptides in chronic heart failure. Card Fail Rev 2019;5:44–49. 30847245 10.15420/cfr.2018.26.1 PMC6396059

[20] Anker SD, Koehler F, Abraham WT. Telemedicine and remote management of patients with heart failure. Lancet 2011;378:731–739. 21856487 10.1016/S0140-6736(11)61229-4

[21] Koehler F, Störk S, Schulz M. Telemonitoring of heart failure patients is reimbursed in Germany: challenges of real-world implementation remain. Eur Heart J Digit Health 2022;3:121–122. 36713016 10.1093/ehjdh/ztac017 PMC9707926

[22] Savoldelli A, Regazzoni V, Rizzola G, Giudici V, Vitali A, Regazzoni D, et al Telemedicine and remote management of patients with heart failure: from theory to daily practice. Telemed J E Health 2024;30:2620–2629. 38963767 10.1089/tmj.2024.0067

[23] Umeh CA, Torbela A, Saigal S, Kaur H, Kazourra S, Gupta R, et al Telemonitoring in heart failure patients: systematic review and meta-analysis of randomized controlled trials. World J Cardiol 2022;14:640–656. 36605424 10.4330/wjc.v14.i12.640 PMC9808028

[24] Koehler M, Funk-Hilsdorf TC, Koehler F. Telemedicine in chronic heart failure. Inn Med (Heidelb) 2025;66:1047–1058. 40879721 10.1007/s00108-025-01969-3

[25] Koehler F, Hindricks G. Is telemonitoring for heart failure ready after a journey longer than two decades? Eur Heart J 2023;44:2927–2929. 37366251 10.1093/eurheartj/ehad395

[26] Ong MK, Romano PS, Edgington S, Aronow HU, Auerbach AD, Black JT, et al Effectiveness of remote patient monitoring after discharge of hospitalized patients with heart failure: the better effectiveness after transition—heart failure (BEAT-HF) randomized clinical trial. JAMA Intern Med 2016;176:310–318. 26857383 10.1001/jamainternmed.2015.7712 PMC4827701

[27] Chaudhry SI, Barton B, Mattera J, Spertus J, Krumholz HM. Randomized trial of telemonitoring to improve heart failure outcomes (Tele-HF): study design. J Card Fail 2007;13:709–714. 17996818 10.1016/j.cardfail.2007.06.720 PMC2702538

[28] Chaudhry SI, Mattera JA, Curtis JP, Spertus JA, Herrin J, Lin Z, et al Telemonitoring in patients with heart failure. N Engl J Med 2010;363:2301–2309. 21080835 10.1056/NEJMoa1010029 PMC3237394

[29] Ziegler A, Öner A, Quadflieg G, Betschart RO, Thiéry A, Babel H, et al Cost-effectiveness of a telemonitoring programme in patients with cardiovascular diseases compared with standard of care. Heart 2023;109:1617–1623. 37316165 10.1136/heartjnl-2023-322518 PMC10579463

[30] Boyne JJJ, Di Van Asselt A, Gorgels APM, Steuten LMG, De Weerd G, Kragten J, et al Cost–effectiveness analysis of telemonitoring versus usual care in patients with heart failure: the TEHAF–study. J Telemed Telecare 2013;19:242–248. 24163233 10.1177/1357633X13495478

