# Supplementary material for: Structuring telemonitoring in heart failure care in The Netherlands: design and operational protocol of a nationwide initiative
Source: Eur Heart J Digit Health. 2025 Nov 5;7(2):ztaf130. doi: 10.1093/ehjdh/ztaf130 (PMC12853108; doi:10.1093/ehjdh/ztaf130)
Supplement: ztaf130_Supplementary_Data [file ztaf130_supplementary_data.docx]

Table of contents supplementary materials

[Supplementary materials 1 – Onboarding process 2](#_Toc208838922)

[Supplementary material 2 – Call scripts 5](#_Toc208838923)

[2.1 Alarm handling 5](#_Toc208838924)

[*2.1.1 Weight: Increase >2kg* 7](#_Toc208838925)

[*2.1.2 Weight: Decrease >2kg* 10](#_Toc208838926)

[*2.1.3 Blood pressure: Hypertension* 11](#_Toc208838927)

[*2.1.4 Blood pressure: Hypotension* 12](#_Toc208838928)

[*2.1.5 Heart rate* 13](#_Toc208838929)

[*2.1.6 Questionnaire: Symptom recognition* 14](#_Toc208838930)

[*2.1.7 Note added* 16](#_Toc208838931)

[*2.1.8 No measurements submitted for too long* 16](#_Toc208838932)

[*2.1.9 Chest pain* 17](#_Toc208838933)

[*2.1.10 Assess urgency* 17](#_Toc208838934)

[2.2 Diuretics protocol workflow 19](#_Toc208838935)

[Supplementary materials 3 – The role of NT-proBNP 22](#_Toc208838936)

[Supplementary material 4 – Setting boundaries and generating alarms 23](#_Toc208838937)

[Supplementary materials 5 – Titration scheme 25](#_Toc208838938)

[*a.* *Heart failure with reduced ejection fraction (HFrEF)* 25](#_Toc208838939)

[*b.* *Heart failure with mildly reduced ejection fraction (HFmrEF)* 29](#_Toc208838940)

[*c.* *Heart failure with preserved ejection fraction (HFpEF)* 29](#_Toc208838941)

# Supplementary materials 1 – Onboarding process

General Process for Registering Patients in Luscii for Heart Failure Monitoring in Santeon hospitals, local processes are in place for patient registration in "Zorg bij jou – heart failure" and the installation of the Luscii app. This may be adjusted based on local needs and practices.

Steps for Registering a Patient in Luscii:

1. Check Email Address. Ensure that the patient has a correct and working email address in the Electronic Patient Record (EPD).
2. Check if there are predefined threshold values (as per the Zorg bij jou protocol) for registration. These can be individually adjusted by a level 3 MSC employee if necessary, with appropriate justification.
3. Register the patient either in the EPD or through [Luscii Healthcare Dashboard](https://vitals.luscii.com/nl/healthcare/dashboard) according to local agreements.
4. The patient will receive a confirmation email at the provided email address and can activate their account through the link in the email.
5. The patient should download the “Thuismeten” (Luscii) app from the Play Store or App Store on their smartphone or tablet.
6. Once the app is installed and the patient has activated their account, the app will be active and measurements can begin.
7. Ensure that the patient submits measurements on the designated start date. If the patient fails to do so, contact them and explain the expectations for submitting measurements (weekly for stable patients and daily for unstable patients).

App Installation and Potential Pitfalls for the Patient:

- Searching for the App.
  Instruct the patient to search for the “Thuismeten” app via the Play Store or App Store.
  Additional information on app setup is available on the [Luscii help page](https://kb.luscii.com/nl/knowledge/aan-de-slag-met-de-thuismeten-app).
- Email Address Issues:
  If the app displays a message such as “This email address is unknown, would you like to sign up?” do not proceed with registration. This issue can occur when creating a new registration and can result in incorrect data in the app.
  Double-check the email address. If the issue persists, contact Luscii Support to confirm if the email has been correctly registered.
- Verification Code:
  Once registration is successful, the app will show a screen indicating that a verification code has been sent to the patient’s email.
  The patient must enter this code into the app within 15 minutes for successful registration.
- Final Setup:
  After entering the verification code, the patient must agree to the terms and conditions and allow notifications to receive messages. The app will then display buttons to fill in measurements and answer questions.
- Troubleshooting:
  If the email address is correct but the app still cannot be activated, suggest that the patient uninstall and reinstall the app. This sometimes resolves the issue.
  - If this does not work, contact Luscii Support directly.

Contacting Luscii Support:

- Email: support@luscii.com
  Luscii aims to respond to customer inquiries within 3 business days.
- Phone: 085 13 05 851 (Available Monday through Friday, 8:30 AM - 5:00 PM, excluding holidays).

# Supplementary material 2 – Call scripts

## 2.1 Alarm handling

There are different types of notifications in the program:

1. Weight: Increase >2kg
2. Weight: Decrease >2kg
3. Blood pressure: Hypertension
4. Blood pressure: Hypotension
5. Heart rate
6. Questionnaire: Symptom recognition
7. Note added
8. No measurements submitted for too long
9. Chest pain
10. Assess urgency

**General questions to ask following notifications:**

- We have received a notification that your weight, blood pressure and/or heart rate is/are lower/higher than normal.
- We see in the questionnaire you have completed that you have more complaints than normal.
- Are you experiencing more complaints? What complaints are they exactly? How much have the complaints increased compared to your starting situation? See also the completed questionnaire ‘Recognising complaints’.
- In case of uneasy feelings/doubts: direct contact with the supervisor on duty at the hospital in question about the policy to be implemented.
- Since when have you been experiencing an increase in these complaints?
- Do you have any idea what could be the cause of the increase in these complaints?
- Has anything changed in your medication or diet in the past few days?
- What have you done yourself to alleviate the complaints?
- Have agreements been made with the patient about using extra medication if necessary or temporarily reducing medication?
  - Possible agreements made could be:
    - In case of signs of fluid retention: increase diuretics (usually double)
    - In case of signs of dehydration (below target weight, low blood pressure, dizziness, possible decline in kidney function): reduce diuretics (usually halve)
    - In case of palpitations: if necessary, extra beta blocker (usually also in medication list)
  - If **not**; forward to healthcare provider with request to prepare a treatment plan for patient, make agreements about adjusting medication if necessary and discuss with patient.
  - If **yes**; have you already adjusted your medication according to this agreement?
    - If yes; what is the effect?
    - If not; what is stopping you from taking the extra medication?
      - In case of signs of fluid retention, instruct to actually use the extra medication if necessary.
      - In case of dehydration, instruct to reduce diuretics.
      - Also see the medication list in the electronic patient file (EPD).
- Do you feel flu-like, feverish or ill? Is there nausea and vomiting? Do you have diarrhea? (If yes, how often per day?)

### *2.1.1 Weight: Increase >2kg*

**Complaints that can be reported:**

- Increased fatigue
- Decreased exercise tolerance
- Increased dyspnea
- Orthopnea
- Pitting edema: swollen feet, ankles and legs
- Feeling of fullness in abdomen/swollen abdomen
- Coughing (tickle cough)
- Decreased appetite, nausea, vomiting, which may mask weight gain!
- Nocturia (urinating at night)
- POB
- Palpitations

**Actions to be taken:**

Estimate the degree and severity of complaints. Determine possible cause (or causes) for the complaints:

- Recently (within the past 14 days) reduced diuretic dose (see diuretic escalation protocol if applicable).
  - Find out reason for reducing diuretics (in EPD or in consultation with healthcare provider).
  - If simultaneously titrating up HF medication (ACE inhibitor, ARNI or SGLT2 inhibitor) refer to level 3 MSC staff member (for further optimisation of HF medication).
  - If due to renal dysfunction refer to level 3 MSC staff member.
  - If due to stable on last dosage for a longer period: return to previous dosage.
- Non-compliance with therapy
  - Discuss lifestyle advice, benefits of medication and importance of compliance with therapy.
    - For example: Your HF medication is an important part of good treatment of HF. In addition, you have received advice about the importance of exercise and consciously dealing with salt and fluid. The effects on your symptoms depend mainly on the way you deal with this yourself. If you have difficulty following the lifestyle advice, you can review the education in the app again and contact your HF nurse if necessary.
- Change in cardiac medication by others.
  - Correct causative change by level 3 MSC staff member in consultation with prescriber.
- New comedication such as prednisone, resonium, NSAID.
  - Restore the causative change by the level 3 MSC staff member in consultation with the prescriber.
- Complaints consistent with ischemia (which may also cause a decline in pump function): ask carefully about the nature of the complaints: pressure, stabbing where on the chest, how long present, radiation and presence of vegetative symptoms such as sweating, nausea, pale (face), anxiety and/or restlessness.
  - Familiar with ischemic suffering?
    - Has the patient already used sublingual nitrate?
    - Effective < 5 to 10 minutes: watch
    - Refer to the level 3 MSC staff member in case of recurrence
  - New complaints consistent with ischemia:
    - If at the time of contact there are still complaints, call the GP (in consultation with the patient, by the patient, informal caregiver or MSC employee) for an emergency home visit to the patient, send the EHH with an ambulance for an indication. In case of serious complaints, call 112 (have them called).
    - If free of complaints: consider or, if in doubt, refer to level 3 MSC staff member.
- Arrhythmias (new or increased)
  - Arrange for patient to come in for an electrocardiogram in consultation with Level 3 MSC staff member.
- Decline in kidney function
  - Escalate to Level 3 MSC staff member.
- Infections
  - In patients without valvular disease: advise patient to contact their GP. Pay extra attention to signs of deterioration.
  - In patients with a mechanical valve prosthesis or suspected infection of a pacemaker/ICD pocket: consult with Level 3 MSC staff member.
- Gradual weight gain without symptoms
  - Keep in mind that an alert triggered by exceeding the upper weight threshold means the patient is already more than 2 kg above their target weight. This may be an early sign or a clear indication of fluid retention, even if the patient has not yet reported any symptoms — especially if the weight gain occurred within 3–5 days. This could still be a reason to make a phone call.Weight gain without fluid retention might also be a reason to discuss lifestyle advice with the patient.
  - If the situation is stable and acceptable: forward the alert with a request to adjust the protocol to prevent unnecessary notifications.

Depending on the severity of the weight gain, whether or not accompanied by symptoms, and in case of signs of decompensated HF: temporarily increase (double) the dose of diuretics.
(Refer to the treatment plan if an agreement on this has been made. If no clear agreement exists, escalate to Level 3 MSC staff member. See also the diuretic escalation protocol.)

### *2.1.2 Weight: Decrease >2kg*

**Complaints that can be reported:**

- Dizziness / orthostatic hypotension
- Thirst / dry mouth
- Dry skin and mucous membranes / reduced skin turgor / sunken, dry eyes
- Oliguria
- Constipation
- Nausea / vomiting
- Muscle cramps, especially in hands, legs, and feet
- Headache
- Feeling of weakness
- Reduced physical fitness
- Tachycardia
- Blurred or reduced vision
- Weakness, apathy, delirium (agitation and/or confusion)
- Fever or other signs of infection

**Actions to be taken:**Assess the nature and severity of the symptoms. When is dizziness present? At rest, when standing up or at other times?

Has kidney function been assessed recently?

Determine the possible cause(s) of the reported symptoms:

- Recently increased dose of diuretics:
  - Check renal function and electrolytes in consultation with Level 3 MSC staff member.
  - Temporarily halve (or stop if already on a low dose) the diuretics, depending on the severity of the weight loss and whether symptoms are present. (Refer to the treatment plan if an agreement exists. If not, escalate to Level 3 MSC staff member.)
  - Temporarily suspend any fluid restriction and discuss a limited increase in fluid intake (maximum 250 ml/24 hours extra).
- Gastrointestinal disturbances: diarrhoea, nausea and/or vomiting:
  - Advise the patient to contact their GP if symptoms persist for more than 3 days.
  - Recommend sufficient fluid intake.
  - If weight loss exceeds 2 kg in less than 3 days, escalate to Level 3 MSC staff member.
- Excessive fluid loss due to fever or high external temperature:
  - Advise the patient to stay indoors during hot weather, stay cool, and drink 200 to 400 ml more than usual. If in doubt, escalate to Level 3 MSC staff member.
- In case of infection, see section on weight gain > 2 kg.
- Medication toxicity (e.g. digoxin, amiodarone, antibiotics, NSAIDs, or opioids).
  - Escalate to Level 3 MSC staff member.

### *2.1.3 Blood pressure: Hypertension*

**Definition:**
Blood pressure measured on three separate occasions on three different days, with an average diastolic value >90 mmHg and a systolic value >140 mmHg, under appropriate measurement conditions: a calibrated blood pressure monitor correctly applied and read, with the patient relaxed and seated in the proper position.

- **Optimal blood pressure:** 115/75 mmHg
- **Mild hypertension:** 140–159 / 90–99 mmHg
- **Moderate hypertension:** 160–179 / 100–109 mmHg
- **Severe hypertension:** >180 / >110 mmHg

**Actions to be taken:**

- In cases of previously undiagnosed moderate or severe hypertension, or a sudden increase >30 mmHg: request a repeat measurement.
- If the repeat measurement is also abnormal and/or if values are extremely abnormal and accompanied by symptoms: escalate to Level 3 MSC staff member.
- In case of doubt, consult the Level 3 MSC staff member (preferably by phone).
- If more than three notifications of abnormal values are received that are asymptomatic, escalate to the Level 3 MSC staff member with a request to consider adjusting the threshold values.

### *2.1.4 Blood pressure: Hypotension*

**Definition:**

Blood pressure lower than 90/60 mmHg. Hypotension occurs in more than 15% of HF patients. Preferably measure both in the supine position and while standing. Orthostatic hypotension is a drop in systolic blood pressure (SBP) of ≥20 mmHg and/or in diastolic blood pressure (DBP) of ≥10 mmHg within the first 3 minutes of standing. Orthostatic hypotension occurs in more than 10% of heart failure patients.

**Actions to be taken:**

- Asymptomatic systolic BP ≥90 mmHg: No changes needed.
- Asymptomatic systolic BP between 80–90 mmHg:
  - Investigate non-pharmacological causes such as fever, diarrhoea, etc.
  - If such causes are found, advise GP consultation, with the recommendation *not* to adjust heart failure medication doses—unless in consultation with Level 3 MSC staff.
  - If a pharmacological cause is suspected and BP remains ≥80 mmHg: consider observing and arranging temporary additional BP monitoring.
- Asymptomatic drop of >20 mmHg systolic:
  - If following recent medication adjustment: monitor and observe.
  - If *no* recent medication change: request an additional BP measurement via message.
- Symptomatic systolic BP ≤90–100 mmHg:
  - If symptoms and signs of dehydration are present, refer to the section on weight loss >2 kg.
- If more than three reports of asymptomatic abnormal readings occur:
  - Escalate to Level 3 MSC staff member with a request to consider adjusting the threshold values.

### *2.1.5 Heart rate*

A cardiac arrhythmia may be too fast, too slow, or irregular. The resting heart rate in adults typically ranges between 60 and 100 beats per minute (bpm).

**Bradycardia** = Heart rate ≤ 50 bpm at rest

**Tachycardia** = Heart rate ≥ 100 bpm at rest

**Symptoms that may be reported:**

- Palpitations
- Pounding heartbeat
- Irregular heartbeats
- Chest pain
- Shortness of breath
- Fatigue
- Dizziness (vertigo or blacking out?)
- (Near) collapse
- Concentration and/or memory disturbances
- Additional symptoms may include: sweating, discomfort, anxiety, and nausea

**Actions to be taken:**

- Assess symptoms: How long have they been present? Is the rhythm regular or irregular?
  - If symptomatic, escalate to Level 3 MSC staff member.
- Check whether the patient has an implanted device. Bradycardia is likely a measurement error, especially in cases of known atrial fibrillation.
  - If asymptomatic: Check whether medications (e.g., beta-blockers or antiarrhythmics) have recently been adjusted.
    - If yes: escalate to Level 3 MSC staff member.
    - If no: arrange a follow-up measurement the next day.
  - If symptomatic: escalate to Level 3 MSC staff member.

If more than three alerts are received showing abnormal values without symptoms: escalate to the Level 3 MSC staff member to evaluate whether further action is needed or whether threshold values should be adjusted.

### *2.1.6 Questionnaire: Symptom recognition*

- Shortness of breath:
  - See actions to be taken under *Weight: Increase >2 kg*
- Fatigue:
  - See actions to be taken under *Weight: Increase >2 kg or Weight: Decrease >2 kg*
- Fluid retention (e.g., swelling in abdomen or ankles):
  - See actions to be taken under Weight: Increase >2 kg
- Dizziness:

**Symptoms that may be reported:**

- - Vertigo (spinning dizziness)
  - Lightheadedness or near fainting (presyncope) or fainting (syncope)
  - Unsteady gait, imbalance, or instability while standing

**Actions to be taken:**

In case of loss of consciousness: Call 112 or have 112 called.

Assess the severity and duration of dizziness, tendency to fall, and/or fainting. How long has it been present?

- Is the patient stumbling or tripping?
- Has the patient fallen?
  - Did the patient sense the fall approaching?
  - Has the patient been unconscious?
    - If yes, was the patient incontinent of urine or faeces?
    - Was anyone present who can provide additional details?

Determine the possible cause of dizziness and/or tendency to fall.

- Are palpitations present?
- Does the patient have a device (ICD/CRT) with home monitoring?
  - If yes, ask the patient to send data through Carelink and inform the pacemaker technician that a transmission will follow due to symptoms. They will review this.
  - If the patient does not have home monitoring but has a device: consult with or transfer the patient to the pacemaker technician for further checks or possible device interrogation.
  - Does the patient have a low ejection fraction [<35%] without a device? In consultation with a Level 3 MSC staff member, arrange for the patient to come for an electrocardiogram or, if needed, attend the emergency cardiac unit (ECU) for further examination and possible rhythm monitoring.
- Is there any weight loss and/or signs of dehydration?
  - Follow the *Weight: Decrease >2 kg* protocol.
- Is there orthostasis (symptomatic)?
  - Refer to Level 3 MSC staff member for possible medication adjustment.
- If no worsening symptoms: No action needed.

### *2.1.7 Note added*

Check the importance of the remark and take action if necessary.

### *2.1.8 No measurements submitted for too long*

Send a message via the app requesting that the patient submits measurements today. If no measurements are received, contact the patient (by phone, via an app message, or an e-consult) to inquire about the circumstances and agree that measurements will be submitted as per the protocol.

In the case of absence, the patient can add absence data in the app under 'Settings – Available for Measurements.' If necessary, you can also do this yourself under Patient – Absence. If the patient is admitted, please consider them as absent.

If more than 4 measurement moments are missed despite the above: escalate to the Level 3 MSC staff member with the question of whether the patient should be discontinued (offboarded).

### *2.1.9 Chest pain*

**Actions to be taken:**

- Assess the severity and extent of symptoms.
- Is the pain clearly anginal or is it dyspnoea due to heart failure/decompensation? If there is an increase in dyspnoea without signs of decompensatio cordis, inquire further and refer to the GP if necessary. If in doubt, escalate to the Level 3 MSC staff member.
- Are anginal symptoms still present? See the section on **Ischemia** in case of weight increase.

### *2.1.10 Assess urgency*

1. Always address someone personally on the phone! Verify their personal details (name, address, and contact details).

2. Determine the urgency:

- Follow the normal workflow if the person does not have urgent symptoms.

**Urgency 1:**

- Not ABCDE stable
- Severe shortness of breath (unable to dress/get out of bed due to breathlessness, speaks only a few words between breaths)
- BP < 90/60 mmHg
- Pulse > 140/min

Life-threatening; keep the patient on the phone and call 112 from another device. Afterward, urgently contact the supervising doctor to arrange for patient admission to the emergency department (ED).

**Urgency 2:**

- Rapid onset of shortness of breath
- Severely ill appearance
- Apathy (AVPU not alert, slow response)
- Cold chills (unable to hold a glass of water still)
- Persistent vomiting (multiple times per hour, even on an empty stomach)
- Severe pain
- Vomiting and diabetes
- BP < 110/70 mmHg
- Pulse > 120/min
- Temperature > 42°C after two measurements
- Temperature < 35°C after two measurements

*Urgent, contact a doctor as soon as possible (<1 hour). Likely re-assessment via ED.*

**Urgency 3:**

- Mild shortness of breath (unable to climb stairs)
- Dehydration (insufficient food/fluid intake, little urination, dry mucous membranes)
- Chest pain
- Ill appearance
- Exceeding standard urgent vital parameter thresholds

*Follow-up via phone or see a doctor (triage nurse consults with doctor). Coordinate with the supervisor at a set time.*

## 2.2 Diuretics protocol workflow

**Start of Workflow**

- Weight Gain of 1.5 - 2 kg with No Complaints Reported in the Questionnaire
  *Template message:* Good morning. You have gained weight, but you have not reported any symptoms. For the next 4 days, an extra weight measurement will be scheduled for you. If you experience any symptoms, please contact the Medical Service Centre (or heart failure clinic).

**Actions:**Send the template message: weight gain with *no* symptoms.

- Schedule 4 additional weight measurements in Luscii over the next 4 days.
- If weight is still above the target weight on day 2, contact the patient to assess whether extra diuretics are needed for 2 days instead of 4.
- If the patient reports no symptoms, no additional diuretics are needed.
- If there's uncertainty whether the weight gain is due to decompensation, blood tests should be taken before starting additional diuretics (Kidney function and NT-proBNP) to reduce the risk of acute kidney dysfunction due to incorrect diuretic increase.
- Continue monitoring weight.

Weight Gain *with* Complaints Reported in the Questionnaire

- With 1 symptom, contact the patient and assess whether additional diuretics are needed.
- With 2 or more symptoms, contact the patient and initiate the diuretics protocol.
- Daily template message in Luscii to be selected after each weight measurement and questionnaire for 5 days.
  *Template message*: weight gain with symptoms or signs of heart failure.
  Good morning. You have gained weight. Did you take your extra diuretic tablets as per the instructions yesterday? If not, please take your tablets. Contact the Medical Service Centre (or heart failure clinic) if you have taken your extra diuretics.

**Actions:**

- Send the message.
- Continue with the diuretics protocol.
- Contact the patient in response to the message if they have symptoms.
- Contact the patient if they do not reach out.
- Explain fluid and sodium restriction.
- Wait for the next weight measurement to decide further action in the diuretics protocol.

Weight Loss but Not at Target Weight with Symptoms or Signs of Heart Failure.
*Template message*: Good morning. You have lost weight. You have not yet reached your target weight but are on the right track. Have you experienced less shortness of breath with exertion, rest, or lying down compared to yesterday, and/or less fluid retention? If yes, please take your extra diuretics as instructed and continue following fluid and sodium restriction. If your symptoms have stayed the same or worsened compared to yesterday, please contact the Medical Service Centre (or heart failure clinic).

**Actions:**

- Send the message.
- Continue with the diuretics protocol.
- Contact the patient in response to the message if they have symptoms.
- Contact the patient if they do not reach out.
- Explain fluid and sodium restriction.
- Wait for the next weight measurement to decide further action in the diuretics protocol.

Target Weight Reached, but Symptoms or Signs of Heart Failure Persist
*Template message*: Good morning. You have reached your target weight, but you have reported still having symptoms. Please take your extra diuretics as instructed, and contact the Medical Service Centre (or heart failure clinic) today.

**Actions:**

- Send the message.
- Contact the patient in response to the message if they have symptoms.
- Contact the patient if they do not reach out.
- Ask for more detailed symptom reports.
- Explain fluid and sodium restriction.
- Consult with supervisor for further action. In case of persistent symptoms, perform NT-proBNP testing, and based on the results, decide on further steps such as scheduling a physical consultation or an additional telephone consultation with a cardiologist/heart failure nurse.

Target Weight Reached with No Symptoms or Signs of Heart Failure
*Template message*: Good morning. Good news: You have reached your target weight and have no symptoms anymore. You may resume your normal diuretic dosage.

**Actions:**

- If necessary, contact the patient to explain stopping the additional diuretics and returning to the normal prescribed dosage.
- No standard lab tests are performed after stopping the diuretics escalation protocol.
- Continue with regular follow-up as per the standard heart failure care pathway.

# Supplementary materials 3 – The role of NT-proBNP

With the use of hTMS, NTproBNP plays a central role in the HF care process since it significantly aids in assessing fluid status remotely^16^. Patient specific levels in time have been shown to strongly correlate with volume status^17,18^. NT-proBNP is routinely measured in various stages during the enrolment in the hTMS programme:

1. During onboarding in the context of medication titration, if not been performed recently.
2. When transitioning from a stable to an unstable condition due to symptoms indicative of worsening HF or decompensation.
3. When transitioning from an unstable to a stable condition, e.g. after achieving OMT or, if indicated, following recompensation.
4. At the discretion of a level 3 or 4 MSC employee outside of the aforementioned moments.

Based on these measurements, NTproBNP contributes to assess disease stability and it can be used as an additional parameter if a caregiver is in doubt about a patients’ fluid status (e.g. in the case of weight gain without obvious complaints or vice versa).

# Supplementary material 4 – Setting boundaries and generating alarms

When included in monitoring, the healthcare provider sets the upper and lower weight limits once and adjusts the lower/upper limits of heart rate and blood pressure if necessary. Based on the values, an alarm could be send to the monitoring centre. An orange alarm is generated for 3 or more points (will be explained below). A red alarm requires action to be taken the same working day.

The threshold values ​​mentioned below have been set as standard:

- Weight (measured at the same time/location/scale)
  - Set the threshold value to 2 kg above target weight and 2 kg below target weight
  - Red alarm:
    - Exceeding the threshold value target weight
    - Increase of ≥ 1.5 kg since the last measurement
    - Decrease of ≤ 1.5 kg since the last measurement
- Blood pressure
  - Orange alarm:
    - ≤ 89 mmHg or ≥ 141 mmHg systolic
    - ≤ 49 mmHg or ≥ 89 mmHg diastolic
  - Red alarm:
    - ≤ 79 mmHg or ≥ 181 mmHg systolic
    - ≤ 39 mmHg or ≥ 99 mmHg diastolic
  - In the event of a third orange alarm without complaints and/or required intervention, a nurse specialist will be consulted whether it is necessary to adjust the threshold value for this patient or apply a medical intervention. In the latter case, the patient will be switched to the unstable protocol if he or she was in stable.
- Heart rate
  - Red alarm:
    - ≤ 44 bpm or ≥ 101 bpm
  - To be adjusted individually at the discretion of the cardiologist or nurse specialist, with substantiation.
- Recognizing complaints; an increase in:
  - - Shortness of breath (1 point)
    - Fatigue (1 point)
    - Fluid retention (e.g. swollen abdomen or ankles) (1 point)
    - Dizziness (1 point)
    - Palpitations (1 point)
    - No, I do not have worsening complaints
    - Need to contact a care provider
- In case of three or more points, an orange alarm will be generated.
- Contact the care provider.

The MSC will ask the patient about the complaints in a structured way: call script (supplemental material 2).

# Supplementary materials 5 – Titration scheme

### *Heart failure with reduced ejection fraction (HFrEF)*

This applies to both decompensated and non-decompensated patients.

1. The following protocol is as standardised as possible. It can be deviated from based on haemodynamic parameters, kidney function, and side effects. Additionally, there is a group of patients with significant haemodynamic/biochemical reserve where optimisation might take place faster than the protocol below.
2. If a patient is decompensated, a loop diuretic is the first choice, which can be given in combination with an MRA or an SGLT2 inhibitor.

*Loop diuretic*: furosemide or bumetanide
A. Used in patients with symptoms and signs of congestion to relieve shortness of breath and oedema, regardless of LVEF.
B. Should be used in combination with an ACE inhibitor or ARB and MRA (unless these are not tolerated).
C. Caution is needed with eGFR <30 ml/min, potassium ≤3.5 mmol/L, or systolic blood pressure <90 mmHg.
D. NSAIDs can weaken the effect of diuretics.

*Lab tests should be conducted 7–14 days after starting and after any dose increase (potassium, sodium, urea, creatinine).*

For T=0, labs should be known regardless of LVEF: potassium, sodium, urea, creatinine, NT-Pro BNP.

**T=0**
Start MRA: spironolactone 25 mg / eplerenone 25 mg once daily
A. Can be started immediately with dewatering. In a vulnerable patient or one with high-normal potassium (>4.5), consider starting with 12.5 mg spironolactone.
B. Do not start if eGFR <30 ml/min, potassium >5.0, or systolic blood pressure <95 mmHg.
C. Target dose: Spironolactone 50 mg once daily, Eplerenone 50 mg once daily.

*Lab tests 1 and 4 weeks after start (potassium, sodium, urea, creatinine).*

**T=1**
Start SGLT2 inhibitor: empagliflozin/dapagliflozin 10 mg once daily
A. Fixed dosing, can also be started in cases of fluid overload/decompensation.
B. Do not start if eGFR <20 ml/min (empagliflozin) or eGFR <25 ml/min (dapagliflozin) or systolic blood pressure <95 mmHg (it is known that eGFR may slightly decrease after starting).
C. In type 2 diabetes mellitus, consider lowering oral anti-diabetic medication; refer to a separate local protocol/flowchart or consult the diabetes care provider. Do not start in type 1 diabetes mellitus.
D. Discuss the risk of urogenital fungal infections (1 in 50 to 1 in 100 patients).

*Lab tests 7–14 days after starting (potassium, sodium, urea, creatinine).*

**T=2**
Start ACE inhibitor (captopril/enalapril, lisinopril, ramipril) or ARB: (candesartan, losartan, valsartan) See ESC guidelines for starting doses.
A. Do not start if eGFR <30 ml/min or systolic blood pressure <90 mmHg. If potassium >5.0, be cautious with starting or monitor closely.

*Lab tests 7–14 days after starting (potassium, sodium, urea, creatinine).*

**T=3**
Start beta-blocker: (bisoprolol, carvedilol, metoprolol, nebivolol). See ESC guidelines for starting doses.
A. If there is sinus tachycardia, be cautious about rapidly increasing the dose of this medication, as in severe heart failure, heart rate is needed to maintain cardiac output.
B. Do not start if systolic blood pressure <90 mmHg or heart rate <55/min. Alternative is possibly bisoprolol 1 dd 1.25 mg, carvedilol 2 dd 3.125 mg.
C. Beta-blockers should not be started in patients who are decompensated (can be slowly introduced during the final phase of compensation).
D. In severe heart failure (LVEF <25%), slowly titrate (12.5-25-37.5-50 mg twice daily for short-acting forms) with smaller steps (increase no faster than every 3 days). Target doses: Metoprolol 200 mg once daily (extended release), bisoprolol 5–10 mg once daily, Carvedilol 25 mg twice daily (up to 50 mg twice daily).
E. Do not start if second or third-degree AV block (without pacemaker).
F. In asthma, prescribe a cardioselective beta-blocker.

*Lab tests 7–14 days after starting (potassium, sodium, urea, creatinine).*

**T=4**
Switch ACE inhibitor/ARB to sacubitril/valsartan (24/26 mg twice daily) or titrate ACEi/ARB.
A. Do not start sacubitril/valsartan if eGFR <30 ml/min or systolic blood pressure <95 mmHg.
B. If not tolerated, switch to ACE inhibitor such as enalapril 5 mg once daily, lisinopril 5 mg once daily, or valsartan 40 mg twice daily (with subsequent titration).
C. If switching ACE inhibitor to sacubitril/valsartan, introduce sacubitril/valsartan 36 hours after stopping ACE inhibitor (risk of angioedema); consider switching to an intermediate dose (49/51 mg) if already on a mid to high dose of ACEi.
D. Target dose of sacubitril/valsartan: 97/103 mg twice daily (however, many patients do not reach this dose). Target doses for perindopril: 8 mg once daily, lisinopril: 20–35 mg once daily, enalapril: 20 mg once daily, valsartan: 160 mg twice daily.
E. For entresto (sacubitril/valsartan), a doctor’s declaration is required.

*Lab tests 7–14 days after starting (potassium, sodium, urea, creatinine).*

**Subsequent steps**:
Titrate sacubitril/valsartan to 49/51 mg twice daily and then 97/103 mg twice daily.

Titrate metoprolol to 50 mg once daily, then 75 mg once daily, and 100 mg once daily.
Titrate spironolactone/eplerenone to 50 mg once daily.

A. Consider whether to increase doses based on the start of these medications (see above).
B. These steps are generally not implemented at the same time. The choice is made individually, depending on blood pressure, heart rate, and side effects.
C. Many patients will have already reached the maximum tolerable dose at this stage.
D. Perform lab tests after complete titration of sacubitril/valsartan or spironolactone.

**Additional steps**:
If stable symptomatic HFrEF with sinus rhythm and resting heart rate >70 bpm, despite optimal beta-blocker dosing, consider adding ivabradine 5 mg twice daily (up to 7.5 mg twice daily).
Consider reducing loop diuretics (furosemide/bumetanide) during titration, especially with dizziness, low blood pressure, or kidney dysfunction. Attempt to reduce loop diuretics before modifying prognosis-modifying medications. Also, attention must be given to polypharmacy and drug interactions.

1. Heart failure with mildly reduced ejection fraction (HFmrEF)
   For HFmrEF, there is less solid evidence for titrating heart failure medication. However,

various studies in patients with heart failure and LVEF >40% consistently show a (tendency towards) positive effect in this group. Therefore, the most recent guideline suggests considering the start of medication in these patients. Currently, sacubitril/valsartan is not reimbursed for this patient group. The titration of patients with HFmrEF should follow the previously established titration protocols, but instead of sacubitril/valsartan, an ACE inhibitor or ARB should be chosen. The most evidence in this group is for SGLT2 inhibitors and aldosterone antagonists (spironolactone/eplerenone).

### *Heart failure with preserved ejection fraction (HFpEF)*

**T=0**
Start SGLT2 inhibitor: empagliflozin/dapagliflozin 10 mg once daily.
A. Fixed dosing, can also be started in cases of fluid overload/decompensation.
B. Do not start if eGFR <20 ml/min (empagliflozin) or eGFR <25 ml/min (dapagliflozin) or systolic blood pressure <95 mmHg (it is known that eGFR may slightly decrease after starting).
C. In type 2 diabetes mellitus, consider lowering oral anti-diabetic medication; refer to a separate protocol/flowchart. Do not start in type 1 diabetes mellitus.
D. Discuss the risk of urogenital fungal infections (1 in 50 to 1 in 100 patients).

*Lab tests 7–14 days after starting (potassium, sodium, urea, creatinine).*

**T=1**
Start MRA: spironolactone 25 mg / eplerenone 25 mg once daily.
A. Can be started immediately with dewatering. In a vulnerable patient or one with high-normal potassium (>4.5), consider starting with 12.5 mg spironolactone.
B. Do not start if eGFR <30 ml/min, potassium >5.0.
Target dose: spironolactone 50 mg once daily, eplerenone 50 mg once daily.

*Lab tests 1 and 4 weeks after start (potassium, sodium, urea, creatinine).*

**Other considerations**:
It is important that in patients with HFpEF (as with all heart failure patients) euvolemia is pursued. Diuretic optimisation should be done in all these patients based on their filling status.
Furthermore, the effect of the aforementioned medication is less effective than in HFrEF, and it is essential to optimise blood pressure, filling status, and lifestyle in patients with HFpEF, with specific attention to possible weight reduction and exercise.

**Note 1**: This medication works better with low-normal LVEF than with supranormal LVEF.
**Note 2**: For hypertension in HFpEF, an ACE inhibitor or ARB is preferred over a calcium antagonist.
